# Time‐encoded golden angle radial arterial spin labeling: Simultaneous acquisition of angiography and perfusion data

**DOI:** 10.1002/nbm.4519

**Published:** 2021-05-03

**Authors:** Merlijn C. E. van der Plas, Sophie Schmid, Maarten J. Versluis, Thomas W. Okell, Matthias J. P. van Osch

**Affiliations:** ^1^ C. J. Gorter Center for High Field MRI, Department of Radiology Leiden University Medical Center Leiden the Netherlands; ^2^ Leiden Institute of Brain and Cognition (LIBC) Leiden University Medical Center Leiden the Netherlands; ^3^ Phillips Healthcare Best the Netherlands; ^4^ Wellcome Centre for Integrative Neuroimaging, FMRIB, Nuffield Department of Clinical Neurosciences University of Oxford Oxford UK

**Keywords:** angiography, arterial spin labeling, golden angle radial, magnetic resonance imaging, perfusion MRI

## Abstract

The objective of the current study was to combine a time‐encoded pseudocontinuous arterial spin labeling (te‐pCASL) scheme with a golden angle radial readout for simultaneous acquisition of angiography and perfusion images from one single dataset, both in a highly flexible single‐slice approach as well as within a multislice setting. A te‐pCASL preparation and the golden angle radial readout were both used as a temporal resolution tool to retrospectively choose the temporal window for the reconstruction of both angiography and perfusion images from a single‐slice dataset. The temporal window could be chosen retrospectively and adjusted to the hemodynamics of the volunteer on the scanner for the single‐slice dataset. Angiographic images were reconstructed at a minimum temporal resolution of 69 ms. For the perfusion phase, only the densely sampled center of k‐space was included in the reconstruction. For a multislice acquisition, the golden angle radial readout allowed reconstruction of images with different spatial resolutions to provide angiographic and perfusion information over 10 slices. The te‐pCASL preparation was used as the only source for dynamic information. The multislice acquisition shows the ability of the golden angle radial readout to display the inflow of the labeled blood into the arteries as well as the perfusion in the tissue with full brain coverage. By combining a te‐pCASL preparation with a golden angle radial readout, single‐slice high temporal resolution angiography and good quality perfusion images were reconstructed in a flexible manner from a single dataset. Optimizing the golden angle radial readout for reconstructions at multiple spatial resolutions allows for multislice acquisition.

## Graphical abstract

A time‐encoded pseudocontinuous arterial spin labeling scheme was combined with a golden angle radial readout for simultaneous acquisition of angiography and perfusion images from one single dataset. This allows for retrospectively changing the temporal window, because the golden angle readout was optimized as a temporal resolution tool. Optimizing this readout for different spatial resolutions shows the ability to display the inflow of labeled blood into the arteries as well as the perfusion in the tissue with full brain coverage.

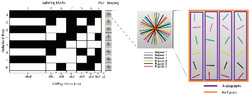

**Abbreviations used**
aBV: arterial blood volume
ASL: arterial spin labeling
ATT: arterial transit time
CBF: cerebral blood flow
MRA: MR angiography
PLD: postlabel delay
pCASL: pseudocontinuous ASL
SNR: signal‐to‐noise ratio
te‐pCASL: time‐encoded pseudocontinuous arterial spin labeling
WM: white matter

## 1 INTRODUCTION

In many cerebrovascular diseases it is of great importance to gain detailed insight into the complete hemodynamic status of the brain. This can be achieved by combining dynamic MR angiography (MRA), which depicts the blood flow in the larger arteries, and perfusion imaging, into a single examination. Whereas the former provides the macrovascular component of cerebral hemodynamics, the latter provides the microvascular viewpoint that ultimately determines the delivery of oxygen and nutrients to brain tissue. Both 4D MRA and perfusion images can be acquired using arterial spin labeling (ASL) MRI,^1^, ^2^, ^3^ which is a noninvasive method that magnetically labels the blood by means of inversion. After subtracting a labeled image in which the inflowing blood is inverted from a control image, and where no labeling of the blood is performed, the contribution of blood to the MR signal is isolated, resulting in images with the desired angiographic or perfusion information. The type of information that is obtained by ASL depends mainly on the postlabel delay (PLD) of the sequence. During the PLD, the labeled blood flows from the labeling plane in the neck to the imaging region in the brain. Choosing a short PLD (≈400 ms) results in angiographic images, whereas perfusion images are acquired using a longer PLD (≈1800 ms^3^). However, acquiring these datasets separately is time‐consuming and thus inefficient, although combined acquisition seems feasible since both depend on a similar labeling module as preparation of the MR signal.

Recently, 4D MRA and perfusion images were acquired simultaneously by Suzuki et al.^4^ A time‐encoded pseudocontinuous ASL (te‐pCASL) module was used to obtain dynamic information. In te‐pCASL the label duration is divided into multiple subboli that are played out in either label or control condition according to a Hadamard matrix.^5^, ^6^ Hadamard decoding during postprocessing can subsequently be used to separate the signal of the different subboli, thereby effectively obtaining ASL images at different PLDs. Since the temporal resolution was already encoded into the labeling, Suzuki et al. only needed a single readout while still obtaining dynamic MRA. This high spatial resolution readout was followed by a spatially more coarse, second readout to capture the perfusion information encoded into the signal by the first subbolus. By only applying small flip angles in the 4D MRA readout, only 20% of the longitudinal magnetization was saturated, preserving 80% of the label (i.e. ASL signal) for the perfusion readout. However, the timing of these MRA images depends on a priori choices of the PLD before data are acquired, that is, before the physiological condition of the subject, and especially the arterial arrival time of the blood, are known.

To overcome these constraints, it was proposed by Okell to combine ASL with a golden angle radial readout to obtain dynamic angiographic and perfusion images from the same dataset.^7^ Radial sampling of k‐space based on the golden angle principle allows for retrospective adaptation of the temporal resolution, since k‐space is uniformly sampled for any arbitrary temporal window.^8^ Moreover, by focusing on the central portion of each spoke that is included in the reconstruction, the effective spatial resolution can be adapted for perfusion imaging. The golden angle radial readout therefore provides an extremely flexible approach to obtain multiple reconstructions from the same data, each with a different trade‐off between spatial and temporal resolution. In this implementation, the golden angle radial readout is the only source of temporal information, thereby necessitating to acquire data over a 2‐s period to cover both the angiographic and the perfusion phase of the label passage. Such a long readout will, however, lead to significant signal attenuation over time of the ASL signal due to the large number of excitation pulses required for covering the dynamic passage of label through the vascular tree. Moreover, to sample with sufficiently high temporal resolution, the approach is best suited to a single‐slice (or limited number of slices) acquisition, although 3D alternatives have recently been proposed.^9^

In the current study, the golden angle radial readout was combined with a time‐encoding (Hadamard) labeling preparation to increase the signal‐to‐noise ratio (SNR), especially during the perfusion phase. The combination of these two sources of dynamic information allows for increased flexibility in sequence design, while in particular enabling fewer excitation pulses and thus higher SNR, both due to less saturation of label, as well as through the option of increasing the flip angle. In addition, by combining these two temporal encoding approaches, single‐slice high temporal resolution angiography and high quality perfusion images can be reconstructed from a single dataset with the possibility to choose the temporal and spatial resolution retrospectively to adapt for the specific hemodynamic state of a subject. Lastly, the golden angle approach can be exploited to reconstruct images at multiple spatial resolutions, which helps in enabling multislice acquisition to achieve whole brain coverage. In this last instance, the time‐encoding labeling scheme will act as the primary source for temporal information and the golden angle approach provides the flexibility in spatial resolution.

## 2 EXPERIMENTAL

Five healthy volunteers (aged 21–34 years, four females and one male) were scanned using a 32‐channel head coil on a 3‐T scanner (Ingenia‐CX; Philips, Best, the Netherlands). Two volunteers were scanned for the single‐slice acquisition, two volunteers were scanned for the multislice acquisition and a single volunteer was scanned for the SNR comparison. All volunteers provided informed consent and the study was approved by the Medisch Etische Toetsingscommissie (METC) Leiden‐Den Haag‐Delft.

### 2.1 SNR comparison

The previous implementation by Okell used a golden angle radial readout with a duration of 2 s to obtain all the temporal information after a single ASL preparation. During these 2 s, 168 spokes were acquired. For the perfusion images, these 168 spokes were divided over eight perfusion frames, each with a temporal window of 252 ms. These 21 spokes were included from all 21 repeated measurements for the reconstruction. Angiographic images contained nine radial spokes from all 21 repeated measurements, which resulted in a temporal window of 108 ms. To limit signal decay due to the large number of excitation pulses that were used during the 2‐s readout, we propose to combine the golden angle radial readout with a Hadamard‐8 labeling scheme. This labeling scheme will provide an additional source of temporal information; therefore, the duration of the golden angle radial readout can be shortened. Less excitation pulses are needed, which does allow the use of higher flip angles for the excitation pulses and thereby results in improved SNR over all time points, which was also shown by Suzuki et al.^10^

To verify whether the proposed combination of the two techniques—the Hadamard‐8 labeling scheme and the golden angle radial readout—actually results in higher SNR during the perfusion phase compared with the method of Okell, simulations were performed, and in vivo data were acquired.

The longitudinal (*M*_*z*_) and transverse (*M*_*xy*_) magnetization were simulated using MATLAB R2019B according to the following formulas:

$$M_{z}\left(t+1\right)=M_{z}\left(t\right)\cdot \cos\left(\alpha \right)$$

$$M_{\mathrm{xy}}\left(t\right)=M_{z}\left(t\right)\cdot \sin\left(\alpha \right),$$

where *t* is a repetition time (TR) index and *α* is the flip angle that was used during the readout, which was 7° for the 2‐s implementation from Okell and 10° for the implementation with the Hadamard‐8 matrix labeling scheme. T_1_‐decay was not included in the simulation since it is the same for both sequences.

**FIGURE 1:**
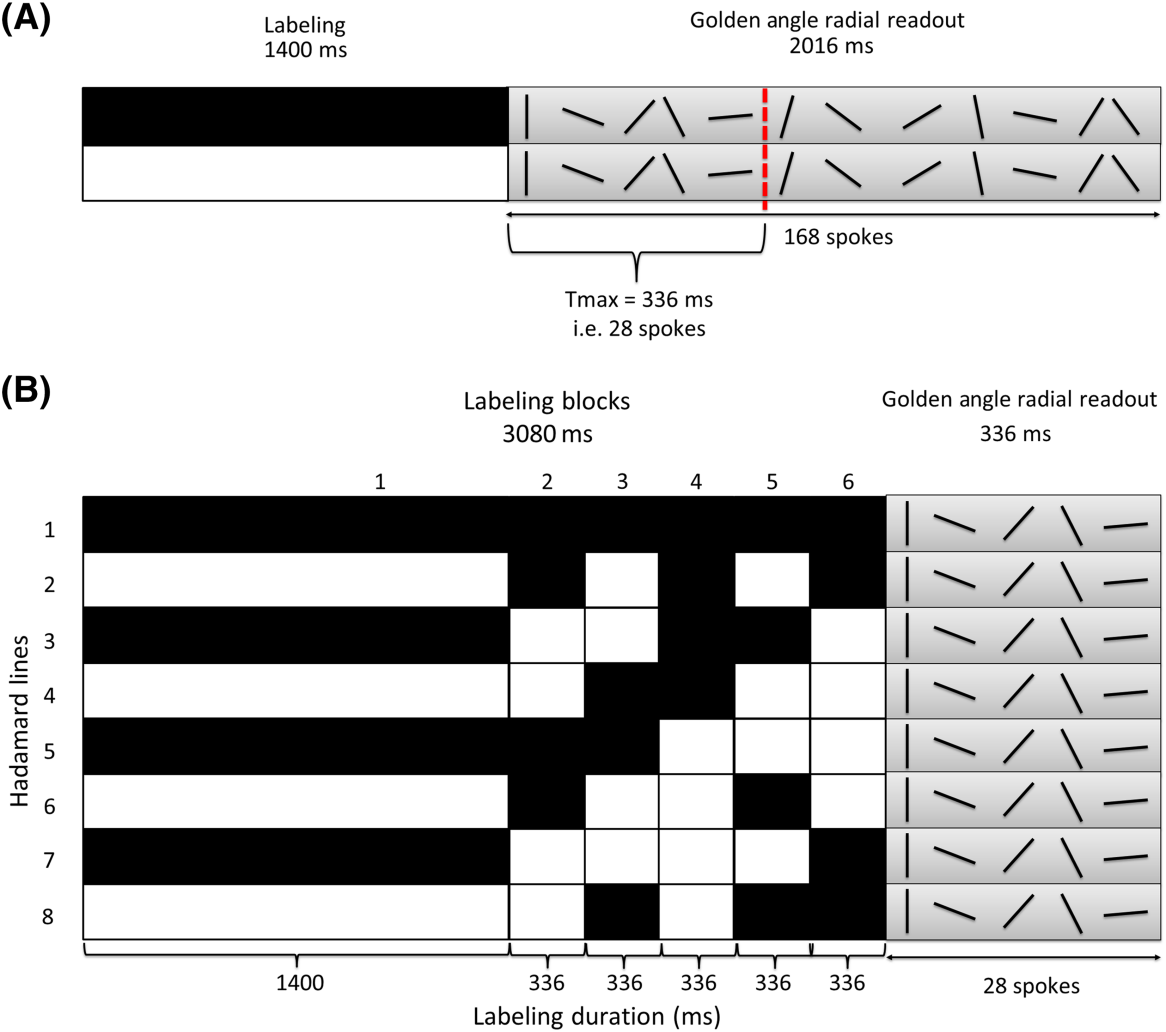
A, Overview of the employed sequence similar to the implementation of Okell. After a label duration of 1400 ms, a golden angle radial readout of 2016 ms starts. Within this 2016 ms, 168 radial spokes are acquired. A maximum temporal window of 336 ms, i.e. 28 spokes is used to order the radial spokes according to the golden angle over the multiple repeats. A flip angle of 7° is used and in total 72 repeats are acquired. B, Overview of the Hadamard‐8 labeling scheme in combination with a golden angle radial readout of 336 ms. The total label duration of the Hadamard matrix is divided over six blocks, therefore the last block duration is set to zero. After the Hadamard preparation 28 spokes are acquired within 336 ms using a 10° flip angle. In total 18 repeats are acquired, since a Hadamard‐8 matrix already provides eight repeats compared with two repeats of a traditional ASL scan

For the in vivo SNR‐comparison, a single‐slice scan similar to Okell's sequence (using an approximation of the t_max_ ordering of the radial spokes; see the next paragraph for an explanation) was compared with our proposed Hadamard sequence. This first sequence had a label duration of 1400 ms, which was directly followed by a golden angle radial readout of 2016 ms (Figure 1A). After every ASL preparation, 168 radial spokes were acquired with 72 repeats, which resulted in a total scan duration of 10 min 34 s. Six perfusion images were reconstructed using a temporal window of 28 spokes (i.e. 336 ms); this way every perfusion image contained 2016 radial spokes consisting of 168 unique radial spokes averaged for 12 repeats. The acquisition voxel size was 1.1 x 1.1 x 10 mm^3^ and the flip angle was 7° (TR/TE = 12/6 ms). Perfusion images were smoothed post hoc with a 3‐mm Gaussian kernel in MATLAB R2019B. This sequence, which has the same essential features as Okell's sequence, was compared with a second sequence, a Hadamard‐8 labeling scheme combined with a golden angle radial readout (Figure 1B). The total label duration of 3080 ms was divided over six blocks: a perfusion block of 1400 ms and five angiographic blocks of 336 ms, which means that the duration of the last block of the Hadamard‐8 matrix was set to zero. These block durations were chosen in such a way that the perfusion block of 1400 ms and its corresponding PLD of 1680 ms would agree with the latest perfusion phase of the sequence, similar to Okell. Since the golden angle radial readout was not the primary source for the temporal information, the readout duration could be minimized to 336 ms. Therefore, less excitation pulses were used, which allowed for an increased flip angle of 10° to be used instead of one of 7°. For the perfusion reconstruction, all the 28 spokes acquired during the 336 ms readout were included. The acquisition voxel size was 1.1 x 1.1 x 10 mm^3^, the total scan duration was 8 min 14 s and TR/TE = 12/6 ms. To keep the total radial spokes within a perfusion reconstruction similar, the Hadamard‐8 labeling scheme was repeated 18 times. This way, all the images after the appropriate subtraction according to the Hadamard matrix also contained 2016 radial spokes (168 unique radial spokes averaged 12 times). Perfusion images were smoothed post hoc with a 3‐mm Gaussian kernel in MATLAB R2019B.

### 2.2 Ordering of the radial spokes within the golden angle radial readout

**FIGURE 2:**
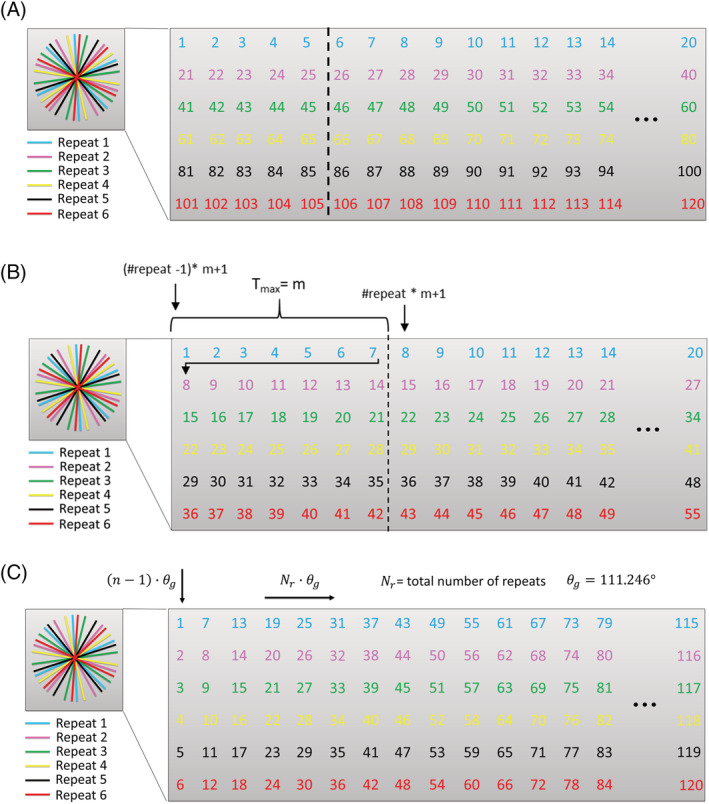
Overview of different ways of ordering radial spokes over multiple repeats. The different colors indicate the different repeats, and the numbers are the consecutive radial spokes according to the golden angle θ_g_. A, Continuous approach; the dotted line indicates a retrospectively chosen temporal window; B, Maximum temporal window approach proposed by Okell; and C, Ordering of radial spokes according to the method described by Song et al. The starting angle of every repeat is determined by: (n − 1) · θ_g_, where n is the repeat and θ_g_ is the golden angle of 111.246°. For every repeat, the increment of the following spoke is equals N_r_ · θ_g_, where N_r_ is the total amount of repeats that will be acquired (assumed to be 6 in this example). Following this ordering will provide the method with full flexibility to retrospectively choose the temporal window for the reconstruction. However, one disadvantage is that when not all repeats are acquired, the true golden angle order is not achieved

The concept of the golden angle radial readout is that post hoc redistributing of k‐lines can provide reconstructions with different spatial or temporal characteristics, while homogenous filling of k‐space is still achieved for the different k‐spaces. The complicating factor for its use in ASL is that multiple encodings (either control/label, or Hadamard time‐encoding) are employed, while one still wants to obtain the freedom to reconstruct images with different temporal and spatial resolution. Therefore, a careful design of the acquisition order of the different spokes is required. In ASL, multiple repeats of the same encoding (either label/control or according to a Hadamard matrix) are averaged to achieve adequate SNR. For golden angle ASL, ordering of the radial spokes across these repeats will determine the uniformity of the coverage in k‐space. Especially for this implementation of the golden angle radial readout, this is very important since for the angiographic images only a few radial spokes per ASL preparation are taken into account for the reconstruction. Multiple methods have been proposed to order the radial spokes according to the golden angle, three of which will be discussed here: (1) the continuous approach, (2) the maximum temporal window approach,^7^ and (3) the approach proposed by Song et al.^11^ (we will refer to this as “Song's ordering”). For the continuous approach, the golden angle order is followed over the multiple ASL preparations that are acquired (Figure 2A). This method will perform very well when all the radial spokes of a single repeat are taken into account for the reconstruction. However, when choosing the temporal resolution retrospectively, indicated by the dotted line, the golden angle order will no longer be fulfilled for this temporal window, since the radial spokes that are included within the reconstructions are not consecutive. This will lead to bigger gaps between neighboring radial spokes than would have been achieved in a true golden angle acquisition, which will eventually result in a decrease in image quality since k‐space is not sampled as uniformly as possible. To remedy this, Okell proposed to use the maximum temporal window (T_max_) approach,^7^ in which the truly golden angle order only holds for this temporal window size, and all the other chosen temporal windows will exhibit inhomogeneous k‐space filling with larger increments between some spokes (Figure 2B). Both previously described implementations limit the flexibility and only achieve a near‐uniform coverage of k‐space under certain conditions. Song et al. proposed a different method to order the radial spokes over the multiple repeats: the starting spoke for repeat *n* is chosen as (*n* − 1) · *θ*_*g*_, with *θ*_*g*_ = 111.246 ° .^11^ For each subsequently acquired spoke, the angle is incremented by *N*_*r*_ · *θ*_*g*_, with *N*_*r*_ the total amount of repeats (Figure 2C). With Song's ordering of the radial spokes, the full flexibility of the golden angle radial readout is attained even when the temporal window is chosen retrospectively. Only when not all of the scheduled repeats can be acquired (i.e. premature ending of the scan) then will the true golden angle order fail. Since we aim for large flexibility regarding the temporal window by combing the Hadamard‐8 labeling scheme with a golden angle radial readout, this particular way of ordering of the radial spokes was used in our single‐slice implementation. The effects of these three different ways of ordering the radial spokes are not evaluated within this manuscript; however, they are discussed to provide an overview of the different methods and to visually demonstrate the theoretical benefits of using a particular ordering within the radial trajectory. Moreover, this highlights that the optimal ordering approach depends on the goal of the golden angle acquisition (i.e. robustness against motion, premature ending of the sequence, or flexibility in temporal and spatial resolution of the final reconstruction).

### 2.3 Golden angle radial readout as a temporal resolution tool

By exploiting the golden angle radial readout as an additional source for temporal information next to the Hadamard‐8 labeling scheme, high temporal resolution angiographic images as well as low temporal resolution perfusion images can be reconstructed from a single dataset. Preferably, angiographic images are reconstructed at a high temporal and high spatial resolution to visualize the labeled blood traversing the arteries. This high temporal resolution can be achieved since angiographic images are sparse, which allows for an undersampled k‐space. In addition, the SNR at the short PLDs is high since all the labeled blood is concentrated within the arteries, while the label has not experienced a lot of longitudinal relaxation yet. Therefore, only a few radial spokes per ASL preparation are included for reconstructions during the angiographic phase to provide a high temporal resolution. At the later PLDs, during the perfusion phase, a lower temporal and spatial resolution is warranted to compensate for the lower SNR originating from the dilution of the labeled blood over the complete tissue compartment and loss of label due to T_1_‐relaxation. To also achieve an adequate SNR during the perfusion phase of the signal, more averaging of signal by including more radial spokes is required, which will consequently lead to a broader temporal window. Such a more coarse temporal resolution is not problematic since the dynamic changes in signal are slower in the perfusion phase compared with the angiographic phase. A low spatial resolution was achieved by only using the densely sampled center of k‐space for the reconstruction.

**FIGURE 3:**
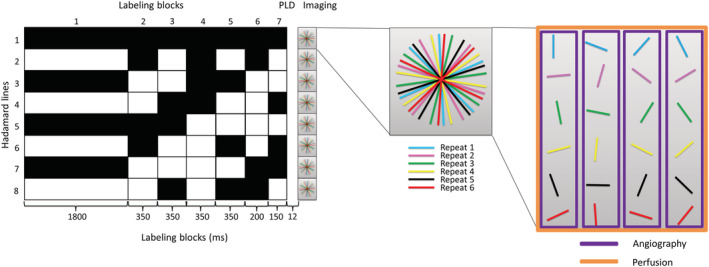
Overview of the Hadamard‐8 labeling scheme for a single‐slice turbo‐field echo (TFE) single‐shot golden angle radial readout. The total label duration of 3550 ms was divided over seven subboli of 1800, four x 350, 200 and 150 ms. A minimum PLD of 12 ms was used. The black blocks indicate labeling; the white blocks indicate control condition, and the numbers below the blocks represent the duration in milliseconds. Background suppression pulses were played out at 1850 and 3130 ms after start of labeling

To obtain high temporal resolution angiography in combination with perfusion images from a single dataset, the golden angle radial readout was used as a second source for temporal information next to the time encoding, since it allows for retrospectively changing the temporal resolution by including a particular set of spokes in the reconstruction while still maintaining an almost uniform coverage of k‐space. To this end, the readout was limited to a single‐slice acquisition with a slice thickness of 12 mm (Figure 3). Since the coarse temporal information is encoded within the Hadamard labeling scheme, the golden angle radial readout duration could therefore be minimized to 346.3 ms with fewer excitation pulses, as in the Okell approach; this is similar to the approach undertaken in the SNR comparison experiments. The total label duration of 3550 ms was divided into seven subboli of 1800, 350, 350, 350, 350, 200 and 150 ms with a minimum PLD of 12 ms, which was followed by a turbo‐field echo (TFE) single‐shot golden angle radial readout with flip angles of 10°. For background suppression, two frequency offset corrected inversion (FOCI) pulses were inserted at 1850 and 3130 ms; since these FOCI pulses were interleaved with the Hadamard labeling, switching between label and control took place after each inversion pulse. The total scan duration was 8 min 21 s, during which 16 repeats were acquired. The voxel size was 0.78 x 0.78 x 12 mm^3^, the matrix size was 256 x 256, an undersampling factor of four was used and the TR/TE were 5.5/2.5 ms. The angiographic images were reconstructed with an effective matrix of 256 x 256, whereas the perfusion images were reconstructed with a 70 x 70 matrix (i.e. using only the densely sampled center of k‐space). A Tukey window, with a roll‐off factor of 10, was used to prevent Gibbs ringing.^12^ The temporal resolution was adjusted by taking the hemodynamics of the volunteer into account; this way the minimum temporal window of the reconstruction could be smaller for a volunteer with a fast blood flow and could be larger for a volunteer with a slower blood flow. This choice can be made based upon a first reconstruction performed after Hadamard subtraction. The scanner reconstruction software RECON2.0 (Philips Healthcare, Best, the Netherlands), which uses a regridding algorithm,^13^ was used for retrospective reconstructions on the scanner.

### 2.4 Golden angle radial readout as a spatial resolution tool

**FIGURE 4:**
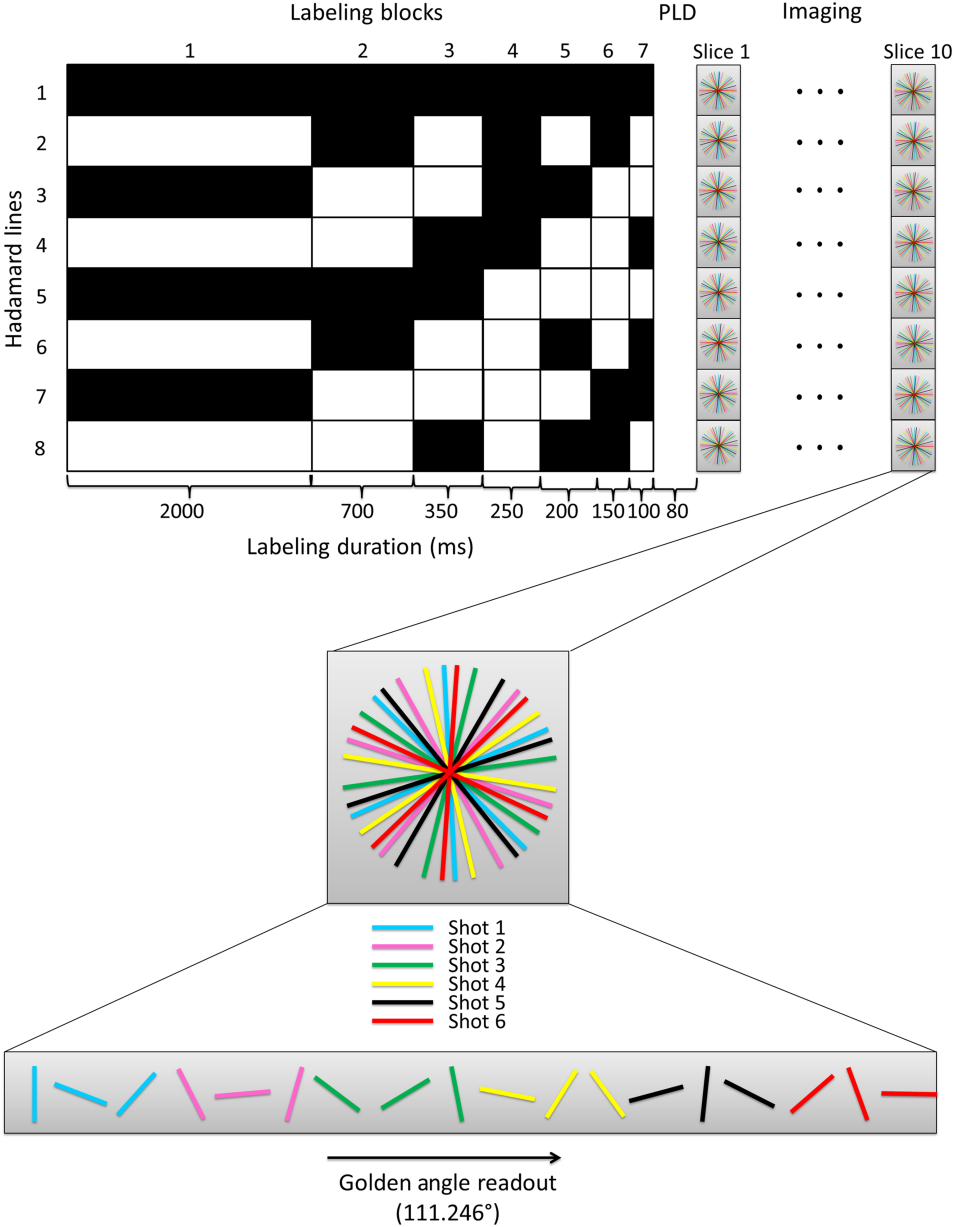
Overview of the proposed sequence were the golden angle radial readout is optimized as a spatial resolution tool, which enables multislice acquisition. The Hadamard‐8 labeling scheme, which will provide the temporal information, is followed by a turbo‐field echo (TFE) multishot golden angle radial readout. The total label duration of 3750 ms was divided over seven subboli of 2000, 700, 350, 250, 200, 150 and 100 ms. A minimum PLD of 80 ms was used and 10 slices were acquired after each Hadamard preparation. The black blocks indicate labeling; the white blocks indicate control condition, and the numbers below the blocks represent the duration in milliseconds. Background suppression pulses were played out at 1950 and 3300 ms after start of labeling

By using the Hadamard 8‐matrix as the only source for the temporal information, the golden angle radial readout could be optimized as a spatial resolution tool, which is particularly useful for multislice acquisition. The readout duration is minimized to 71.4 ms, which allows for the acquisition of several slices after every Hadamard preparation. Since the readout duration was minimized, fewer excitation pulses were used compared with the single‐slice acquisition and therefore the flip angle could also be increased to 20° to improve SNR. To obtain both angiographic signal at a high temporal resolution and perfusion images with sufficient SNR within this implementation, the block durations of the Hadamard labeling scheme, which dictate the temporal resolution, were chosen to be 2000, 700, 350, 250, 200, 150 and 100 ms. In combination with a minimum PLD of 80 ms, this results in angiographic images with a high temporal resolution during the earlier PLDs. During the perfusion phase at the later PLDs of 1130 and 1830 ms, the label durations were chosen as 2000 and 700 ms to obtain sufficient SNR. Two FOCI pulses at 1950 and 3300 ms were used for background suppression. The ASL preparation was followed by a multishot acquisition. For this multishot acquisition multiple radial spokes were acquired after a single ASL preparation; this way k‐space was filled according to the golden angle in 16 shots of 71.4 ms (Figure 4). For every shot that contained a particular section (i.e. 16 spokes, matrix of 256) of the total amount of spokes, the complete Hadamard matrix was acquired. In a total scan time of 9 min 39 s, 10 slices were acquired to obtain whole brain coverage. A voxel size of 1.25 x 1.25 x 10 mm^3^ was used and TR/TE of 4.4/1.95 ms. The perfusion phases, which are determined after the appropriate Hadamard subtraction based on the hemodynamic condition of the volunteer, of this multislice dataset were smoothed post hoc using a Gaussian filter in MATLAB R2019B.

These multislice datasets were quantified after subtraction according to the appropriate Hadamard matrix. The BASIL toolkit of the Oxford Centre for Functional MRI of the BRAIN (FMRIB)’s software library (FSL) was used to quantify the ASL signal within a probabilistic analysis approach.^14^, ^15^, ^16^ Within this framework the macrovascular contribution to the ASL signal can be fitted and separated from the perfusion signal using the two‐component kinetic model. This results in cerebral blood flow (CBF), arterial blood volume (aBV) and arterial transit time (ATT) maps. A single M0 value, which was determined manually in a region of interest located in the white matter (WM) by visual inspection, was used for calibration after correction for the brain partition coefficient of water in the WM (λ = 0.82) and corrected for longitudinal relaxation.^17^

## 3 RESULTS

### 3.1 SNR comparison

**FIGURE 5:**
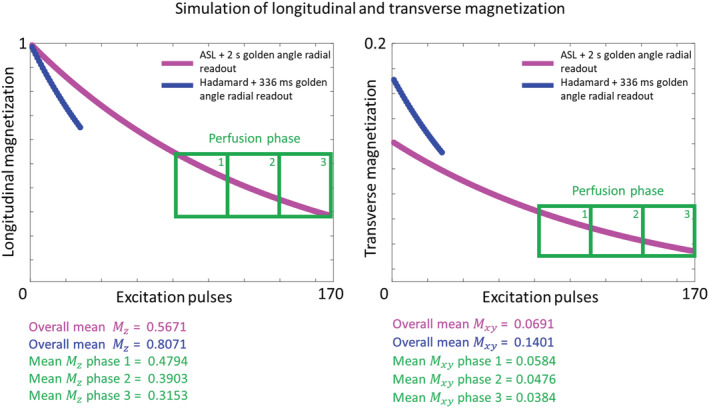
Results of the longitudinal and transverse magnetization simulations for the proposed implementation, which combines the golden angle radial readout with a Hadamard preparation, and a 2 s golden angle radial readout

Figure 5 shows the simulation results for the longitudinal and transverse magnetization for the ASL with the 2‐s readout according to the method of Okell and for the proposed implementation of the golden angle radial readout in combination with a Hadamard‐8 labeling scheme. The overall mean transverse magnetization is more than two times higher for the proposed implementation. Moreover, since the perfusion phase of the signal is at the end of the 2‐s golden angle radial readout (indicated by the green boxes in Figure 5), the transverse magnetization increased with a maximum of factor 3.6 for the proposed implementation.

**FIGURE 6:**
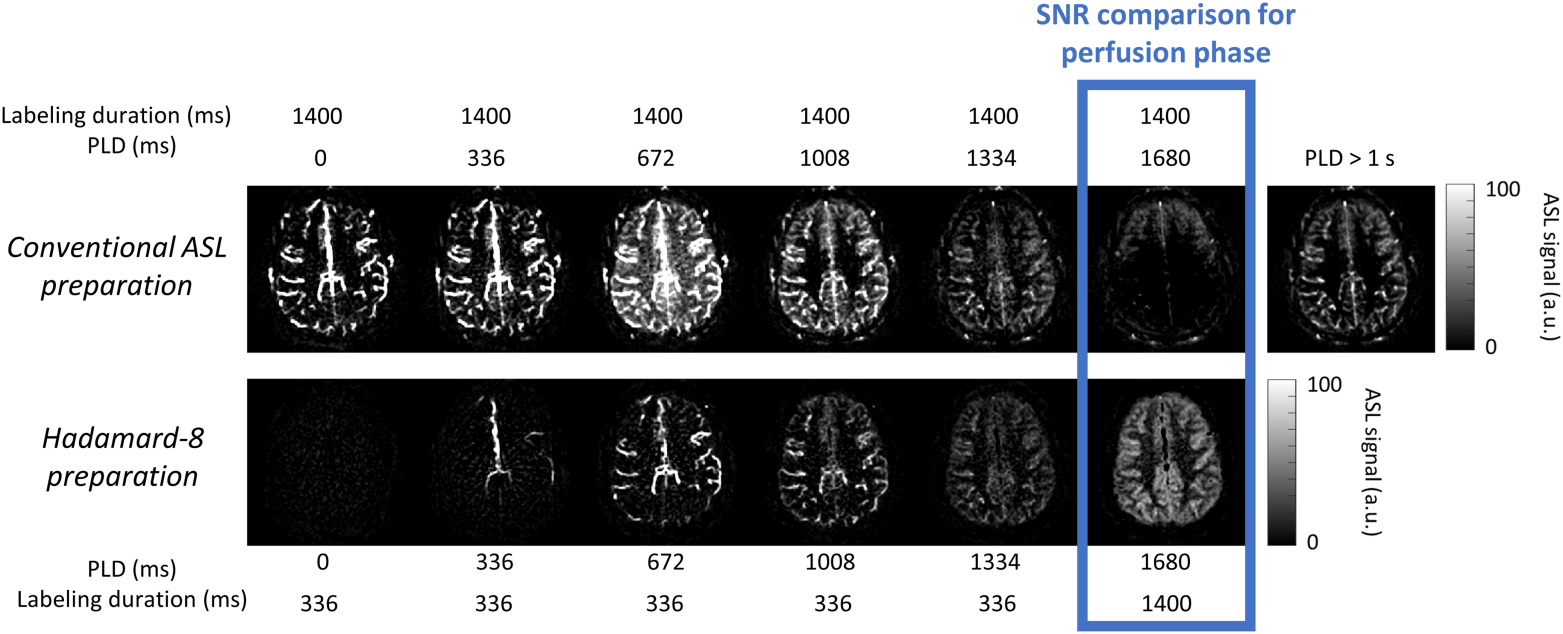
Qualitative comparison of single‐slice acquisition between a scan similar to the sequence of Okell and the proposed sequence, which combines a Hadamard labeling preparation with a golden angle radial readout. The top row shows the results for the six different perfusion frames with a temporal window of 336 ms. Due to the many excitation pulses that were used, signal attenuation over time was present. Therefore, a temporal mean of all PLDs above 1 s was shown, as was also done in Okell. The bottom row shows the six PLDs for the proposed sequence. Since the Hadamard preparation was also used for temporal information, the readout duration was only 336 ms. Therefore, less excitation pulses were needed, which led to more signal during the perfusion phase at a PLD of 1680 ms

Figure 6 shows the in vivo comparison of both the described techniques. The top row shows the six perfusion frames for the sequence similar to the sequence of Okell, where 168 radial spokes were acquired sequentially in 2016 ms after a pCASL preparation of 1400 ms. Since many excitation pulses of 7° were used, signal attenuation over time is present, and which was observed for the PLDs of 1334 and 1680 ms. For the results of Okell the temporal mean of all PLDs above 1 s was taken; this averaged perfusion image is also shown in Figure 6. For the sequence that combined the golden angle radial readout with a Hadamard‐8 labeling preparation, a shorter readout duration of 336 ms could be used in which less excitation pulses are used. The SNR comparison was mostly focused on the perfusion phase at PLD 1680 ms, which provides a fair comparison because label duration and PLD are equal for both approaches. Image quality and SNR are clearly better for the Hadamard preparation compared with the conventional approach. Note that in the Hadamard implementation, the angiography images are obtained from blocks with a much shorter labeling duration than the 1400 ms traditional ASL preparation of the first row. Therefore, images show label more contained in arteries, as opposed to combined perfusion and angiography information in the first sequence. Temporal SNR (tSNR) for this perfusion phase at a PLD of 1680 ms was calculated as follows: $\text{tSNR}=\frac{\text{mean signal}}{\text{stdev over time}}$ and showed an overall increase of tSNR when the golden angle radial readout was combined with a Hadamard‐8 preparation (see Supplementary Data S1 for the tSNR‐maps for both implementations). The shortest PLD for the Hadamard sequence did not show any signal, since the labeled blood had not yet arrived at the imaging plane.

### 3.2 Golden angle radial readout as a temporal resolution tool

**FIGURE 7:**
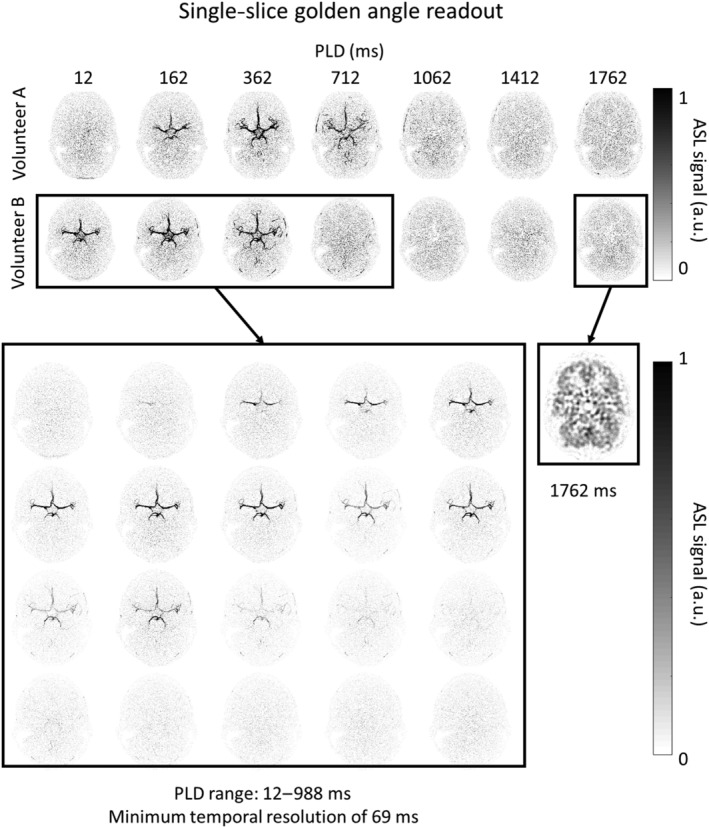
Single‐slice ASL dataset after the Hadamard subtraction reconstructed at the timepoints of the Hadamard labeling scheme. Since the blood flow of both volunteers, but especially volunteer B, are fast compared with the temporal windows that are determined by the Hadamard labeling scheme, the in‐ and outflow of the labeled blood within the bigger arteries is not displayed accurately. Therefore, the same data of volunteer B were retrospectively reconstructed at the minimum temporal resolution of 69 ms. This reconstruction shows the in‐ and outflow of the labeled blood within the bigger arteries. The perfusion phase at a PLD of 1762 was reconstructed using only the densely sampled center of k‐space

Figure 7 shows two examples of the single‐slice datasets after Hadamard subtraction. The blood flow of volunteer B is very fast, therefore the inflow of the labeled blood in the bigger arteries is not shown when the temporal information was only determined by the Hadamard‐8 matrix. However, since the golden angle radial readout was used, the temporal window could be adjusted to the specific hemodynamic condition of this volunteer. It was therefore decided to reconstruct the angiographic phase for volunteer B at a minimum temporal resolution of 69 ms. The results of this reconstruction, which now clearly depicts the in‐ and outflow of the labeled blood in the bigger arteries correctly (compared with the top images), are shown in the bottom part of Figure 7. The perfusion phase at a PLD of 1762 ms was reconstructed using only the densely sampled center of k‐space (70 x 70 matrix).

### 3.3 Golden angle radial readout as a spatial resolution tool

**FIGURE 8:**
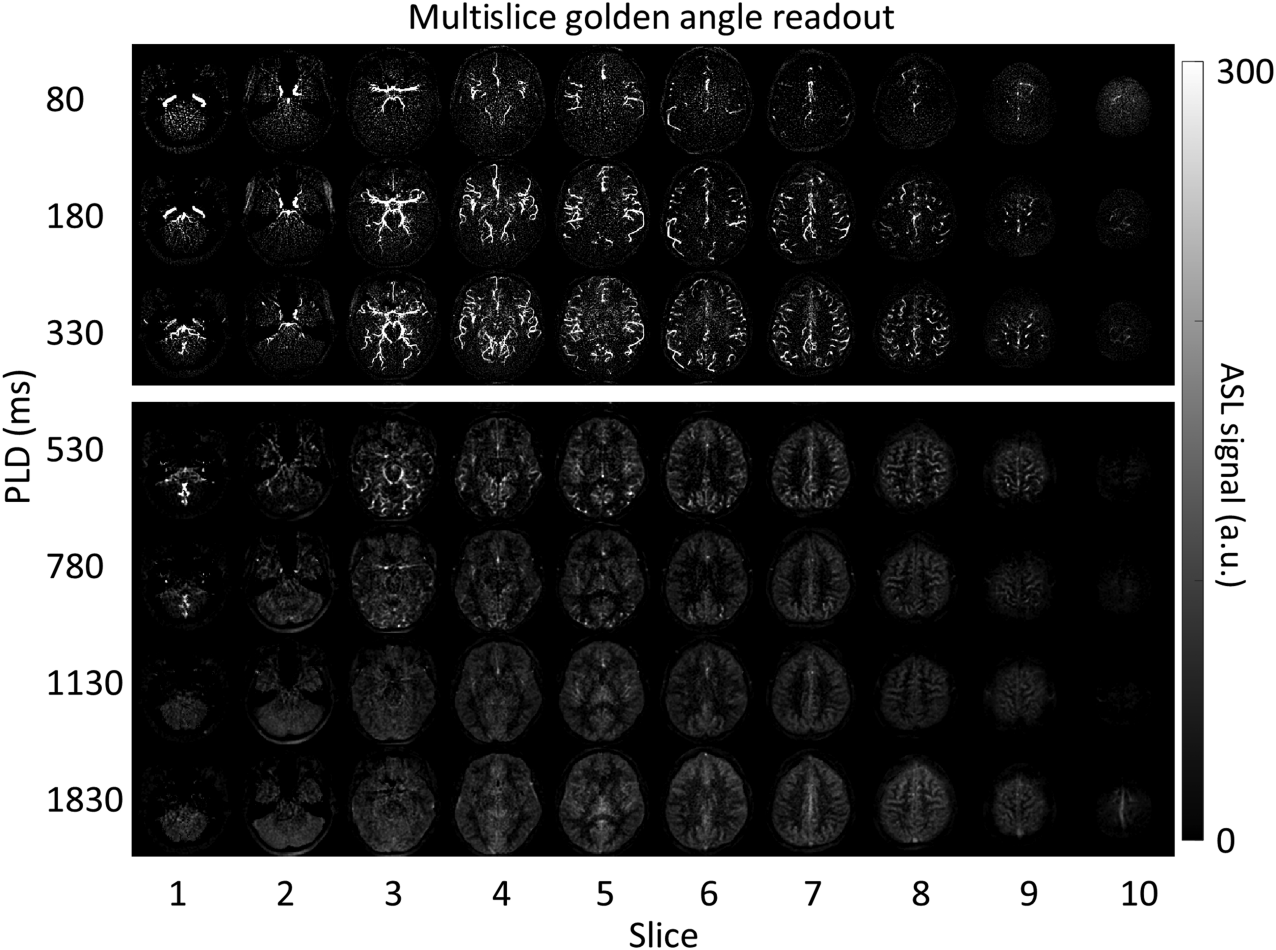
Subtracted ASL signal at seven postlabeling delay time points for 10 slices. The top part shows the angiographic phases and the bottom part shows the perfusion phase, which was smoothed post hoc using a Gaussian filter in MATLAB

Figure 8 shows the subtracted multislice ASL data for a representative volunteer. To enable whole brain coverage, the golden angle radial readout was optimized as a spatial resolution tool. This way, the Hadamard‐8 labeling scheme acted as the primary source for the temporal resolution, which resulted in PLDs of 80, 180, 330, 530, 780, 1130 and 1830 ms. The top part of Figure 8 shows the angiographic phase reconstructed at high spatial resolution, where the labeled blood traverses through the macrovasculature. Lower spatial reconstructions for displaying the perfusion phase (PLD > 572 ms) are shown in the bottom part. These reconstructions were made by post hoc smoothing using a Gaussian filter in MATLAB R2019B.

**FIGURE 9:**
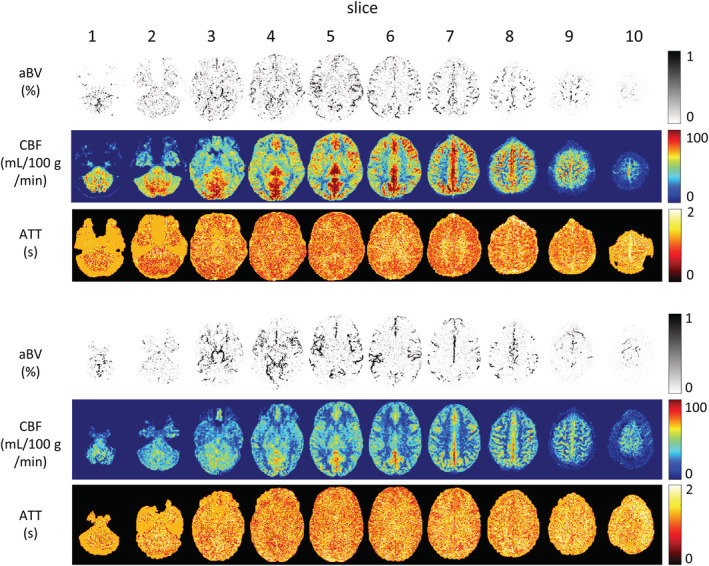
CBF‐, ATT‐ and aBV‐maps for two volunteers estimated for the multislice dataset. The BASIL toolkit was used to quantify the acquired data, with the first row showing the aBV‐map including the macrovascular contribution in the Bayesian analysis. The ATT‐ and CBF‐maps are shown in the second and third row, respectively. A shows the quantified results for the dataset that is shown in Figure 8

Figure 9 shows the quantitative results for the two multislice acquisitions. Since a more complex model was used for the quantification, the macrovascular component was isolated from the perfusion component resulting in aBV‐ (top row), CBF‐ (middle row) and ATT‐ (bottom row) maps. The aBV‐maps show the macrovascular contribution to the ASL signal. Both CBF‐maps show the usual gray matter (GM) perfusion pattern, although the CBF values of volunteer C are higher compared with the CBF values of volunteer D. The ATT‐maps demonstrate higher ATT values in the WM compared with the GM.

## 4 DISCUSSION

In this study the golden angle radial readout was combined with a Hadamard‐8 labeling scheme to explore the opportunities for combined acquisition of angiographic and perfusion images within a single ASL sequence. The most important benefits of this approach are threefold: increased SNR, enhanced flexibility and improved ability for multislice acquisition.

First, using two sources for obtaining temporal information (i.e. the golden angle radial approach and Hadamard encoding), higher SNR was achieved during the perfusion phase compared with the original golden angle approach, as proposed by Okell. Since the Hadamard‐8 labeling scheme could act as a prime source of temporal information, the golden angle radial readout could be minimized to 346.4 ms. This led to a decrease in the number of excitation pulses that were used and thus less saturation of label signal, which in particular had a large impact on the SNR of the later phases. Therefore, the flip angle could also be increased, which led to an even higher SNR. This was demonstrated with simulations in Figure 5 and with an in vivo scan in Figure 6. An increase of signal is shown for a PLD of 672 ms in the top row of this figure, which could be caused by the lack of background suppression in this acquisition, the occurrence of motion, or by the fact that most signal is present at this particular time point. The SNR comparison was mostly focused on the perfusion phase at a PLD of 1680 ms. A limitation of the simulations is that T_1_‐ and T_2_‐decay were neglected based upon the argument that they will have similar effects for both sequence options. It is therefore assumed that these simulations still provide a proper reflection of how SNR is affected when using these two different sequences. It should be noted that, in general, it is crucial to take T_1_‐ and T_2_‐decay into account when determining the optimal flip angle, especially when a long readout with many excitation pulses is used.

Second, by implementing the ordering of the radial spokes according to the method that Song et al. proposed, a true golden angle order over the multiple repeats was obtained even when the temporal window was chosen retrospectively. The reconstruction of the proposed method was implemented in the RECON2.0 software, which allowed for retrospective reconstructions of the images with different temporal sampling patterns on the scanner itself, allowing easy integration into the clinical workflow. Therefore, the images can be reconstructed right after the scan is acquired and the temporal window can be chosen retrospectively and adjusted to the specific hemodynamics of the subject, which would be beneficial for patients with, for example, an increased arterial arrival time. This was demonstrated in Figure 7. It could be considered in the future to use more advanced reconstruction techniques such as an iterative approach and, for example, an AI algorithm to let the scanner automatically select the optimal temporal resolution for every subject that is scanned.

Lastly, since the Hadamard preparation provides the temporal information for this ASL sequence, the golden angle radial readout could also be optimized to provide flexibility in reconstructing ASL images at multiple spatial resolutions in a multislice setting. For this purpose, the readout duration per slice was minimized to 71.4 ms, which allowed for the acquisition of multiple slices after every ASL preparation. This way, angiographic and perfusion images were acquired in 10 consecutive slices, as shown in Figure 8. This figure demonstrates the passage of the labeled blood from the macrovasculature towards the tissue with whole brain coverage. Since the golden angle radial readout was not used as a temporal resolution tool, the temporal window could not be adapted retrospectively for this implementation. Therefore, the durations of the labeling blocks of the Hadamard matrix determine exclusively the temporal information for the acquired images. To be able to achieve more flexibility and to retrospectively choose the temporal window within this whole brain implementation, a 3D kooshball trajectory would be of interest,^18^ although its implementation is more complicated and therefore reserved for future work. Another way to achieve whole brain coverage would be to combine the golden angle radial readout with a simultaneous multislice (SMS) readout.^19^, ^20^ This will increase coverage, minimize relaxation of label in the later acquired slices, and shorten the total readout duration. However, conventional SMS techniques, such as CAIPIRINHA using line‐dependent phase cycling,^21^ do not work for non‐Cartesian sampling schemes with crossing trajectories since they cause destructive interference and loss of signal. Therefore, it has been proposed to combine this line‐dependent phase cycling with intentional destructive interference and effectively reduce the previously coherent signal from an entire slice. This way, radial CAIPIRINHA allows achieving even higher quality compared with Cartesian CAIPIRINHA.^21^

Optimizing the golden angle radial readout as a spatial resolution tool allows for the acquisition of multiple slices and the ability to retrospectively change the spatial resolution for every reconstruction. This can be achieved, for example, by only including the densely sampled center of k‐space in the reconstruction, which will lower the spatial resolution of the images; this could be important, especially for the perfusion phase. In comparison with the more conventional ASL techniques that are using an EPI readout, it was shown by Okell that less dropout and distortion artefacts were present for the radial readout.^7^ It was also shown by Song et al. that when using a 3D radial readout, more signal is present within the more distal arteries compared with a Cartesian readout.^11^ This multislice acquisition was a step towards a 3D readout with increased flexibility compared with 3D segmented EPI ASL acquisition.

In this study, the combination of the two techniques, the golden angle radial readout and the Hadamard‐8 labeling scheme, was used to obtain dynamic ASL signal in both the angiographic and perfusion phases. Using the golden angle radial readout as a temporal resolution tool also enabled adaption of the trade‐off between the temporal and spatial resolution for different reconstructions of the same dataset. This allowed reconstruction of the angiographic phase at a high temporal and spatial resolution, whereas the perfusion phases could be reconstructed at a lower temporal and spatial resolution. Therefore, the data that are acquired with this sequence would also be highly suitable for fitting a kinetic model that differentiates macrovascular from microvascular ASL signal,^14^, ^22^ as well as for more advanced combined reconstruction and analysis approaches that we considered to be beyond the scope of the current study. Multiple parameters such as ATT, aBV and CBF could be extracted from the combined perfusion and angiography data.^23^ This could be useful when studying stroke, occlusive artery disease, Alzheimer's disease and transient ischemic attack,^24^, ^25^ where both microvascular as well as macrovascular pathology might be present.

The use of a Hadamard preparation scheme comes with some limitations. A sequence that employs a Hadamard labeling scheme requires acquisition of more datasets, since the entire Hadamard matrix (in our implementations eight encodings) should be acquired, compared with two encodings (label/control) for conventional ASL‐preparation schemes. For perfusion imaging this is not an issue, since repeated acquisitions (either averaging, more encodings, or shots in a segmented readout) are needed to increase SNR. However, for angiography, where a high spatial and high temporal resolution are required, no repetitions are usually performed, and then Hadamard encoding would lead to longer acquisition durations and/or the need for higher acceleration factors by more undersampling. Especially when moving to 3D, this results in more artefacts, since even more radial spokes should be acquired in 3D to cover k‐space. This will broaden the temporal window of the reconstruction, which results in low temporal resolution angiography images that do not contain as much information as high temporal resolution angiographic images. Due to this broader temporal window, the reconstructions are also more vulnerable to motion. In addition, when using both the Hadamard preparation scheme and the golden angle radial readout as a temporal resolution tool, the use of a constant flip angle of 10° could lead to some signal attenuation, even when the readout duration was decreased to 346.5 ms. This could lead to signal discontinuities when combining spokes from different Hadamard blocks, since the signal from the last spoke from one subbolus will be more highly attenuated than the signal from the first spoke of the next subbolus. Woods et al. showed that the use of a variable flip angle approach could mitigate this problem.^26^

The golden angle radial readout can also be exploited to correct for motion artefacts.^27^, ^28^ Acquiring good quality ASL images usually takes up to around 5 min. For some patient groups it is very difficult to lie still during this acquisition time, which will lead to a loss of the image quality. The golden angle radial readout would enable eliminating the affected radial spokes due to motion from the reconstruction, while keeping an almost uniformly sampled k‐space. Moreover, the fact that the center of k‐space is acquired after each excitation pulse does allow for more advanced reconstruction approaches to correct for motion or other artefacts, such as iterative reconstruction algorithms.

Radial sampling trajectories can be considered rather inefficient sampling schemes since a large number of spokes are required to sufficiently cover k‐space.^9^ Therefore, many excitation pulses are needed, which results in increased SAR values and signal saturation over time. This latter effect is especially detrimental to ASL, due to the fact that the signal is prepared during labeling and therefore no regrowth of signal occurs during the readout. To increase the coverage of k‐space within a single excitation, a spiral sampling trajectory has been proposed.^29^, ^30^ Spiral trajectories are more efficient regarding k‐space coverage and can be made even more flexible by varying the sampling density. Therefore, a variable density spiral readout could even further reduce the readout duration, since the outer part of k‐space could be undersampled. This undersampled outer part of k‐space provides the detailed information for the angiographic images; however, these images are sparse, which makes them rather insensitive to undersampling. The acquisition window per spiral will reduce and, therefore, even higher temporal resolution images can be acquired. Moreover, the center of k‐space could be oversampled, which will increase the SNR of the perfusion images that are reconstructed by only including the center of k‐space. Spiral acquisition could also be combined with Hadamard time‐encoding to achieve similar flexibility, as in our radial approach. By moving to a 3D stack‐of‐spiral readout, which was already combined with a conventional ASL preparation by Chang et al.^31^ it would be possible to acquire multiple time points while maintaining whole brain coverage. To improve tSNR using a spiral readout, it was proposed by Nielsen and Hernandez‐Garcia to combine a segmented low flip angle 3D spiral readout with radiofrequency spoiling and ASL: SNR was improved with a factor of 2 compared with a 2D spiral readout and employing this readout resulted in good quality functional MRI‐images.^32^ Using a golden angle approach to fill k‐space would allow for different reconstructions with each having a different temporal resolution. However, spiral trajectories come with challenges such as off‐resonance issues and gradient trajectory corrections, as well as through slice blurring for a stack‐of‐spiral readout. These challenges should be taken into account when designing the sequence and reconstruction pipeline, for example, by including a conjugate phase reconstruction to overcome off‐resonance effects.^33^

## 5 CONCLUSION

By combining a Hadarmard‐8 labeling scheme with a golden angle radial readout, single‐slice high temporal resolution angiography and good quality perfusion images were reconstructed from a single dataset. The temporal resolution could be chosen retrospectively to adjust it to the hemodynamics of the volunteer. These retrospective reconstructions were performed on the scanner right after the data were acquired. Switching to a multislice acquisition, the Hadamard‐8 matrix was used as the primary source of temporal information. This way the golden angle radial readout could be optimized for reconstructions at multiple spatial resolutions, thereby enabling whole brain coverage.

## Supporting information


**Figure S1.** tSNR‐comparison between two different implementations of the golden angle radial readout for the perfusion phase (label duration of 1,400 ms and PLD of 1,680 ms). Left shows the tSNR‐map for the implementation where a conventional pCASL preparation of 1,400 ms was used. Due to the many excitation pulses used during the readout, the signal was attenuated which results in low SNR. Right shows the tSNR‐map for the Hadamard‐8 preparation in combination with a golden angle radial readout. The readout duration was shorter, since the Hadamard matrix also provided temporal information, and thus the flip angle could be increased to 10°. This resulted in increased SNR during the perfusion phase at PLD = 1,680 ms.
