# Supplementary material for: Time‐encoded golden angle radial arterial spin labeling: Simultaneous acquisition of angiography and perfusion data
Source: NMR Biomed. 2021 May 3;34(7):e4519. doi: 10.1002/nbm.4519 (PMC8243971; doi:10.1002/nbm.4519)
Supplement: Supplementary file 1 — Figure S1. tSNR‐comparison between two different implementations of the golden angle radial readout for the perfusion phase (label duration of 1,400 ms and PLD of 1,680 ms). Left shows the tSNR‐map for the implementation where a conventional pCASL preparation of 1,400 ms was used. Due to the many excitation pulses used during the readout, the signal was attenuated which results in low SNR. Right shows the tSNR‐map for the Hadamard‐8 preparation in combination with a golden angle radial readout. The readout duration was shorter, since the Hadamard matrix also provided temporal information, and thus the flip angle could be increased to 10°. This resulted in increased SNR during the perfusion phase at PLD = 1,680 ms. [file NBM-34-e4519-s001.docx]

*
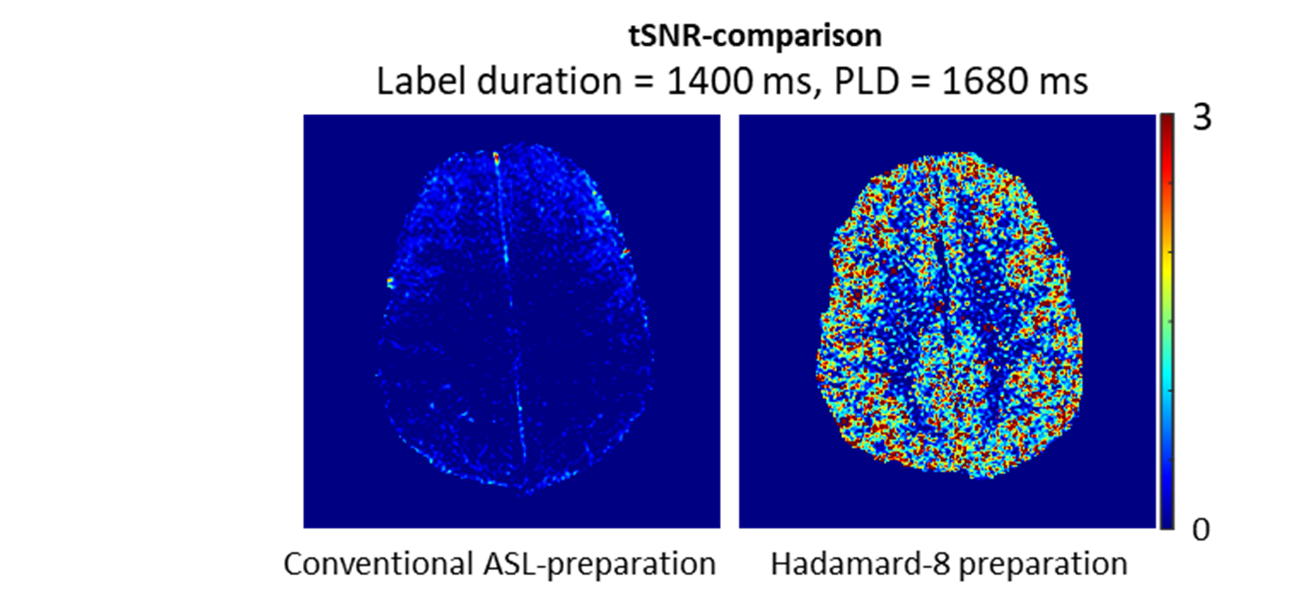
*

*Supplementary figure S1. tSNR-comparison between two different implementations of the golden angle radial readout for the perfusion phase (label duration of 1400 ms and PLD of 1680 ms). Left shows the tSNR-map for the implementation where a conventional pCASL preparation of 1400 ms was used. Due to the many excitation pulses used during the readout, the signal was attenuated which results in low SNR. Right shows the tSNR-map for the Hadamard-8 preparation in combination with a golden angle radial readout. The readout duration was shorter, since the Hadamard matrix also provided temporal information, and thus the flip angle could be increased to 10°. This resulted in increased SNR during the perfusion phase at PLD=1680 ms.*
